# Recommendations for performing, interpreting and reporting hydrogen deuterium exchange mass spectrometry (HDX-MS) experiments

**DOI:** 10.1038/s41592-019-0459-y

**Published:** 2019-06-27

**Authors:** Glenn R. Masson, John E. Burke, Natalie G. Ahn, Ganesh S. Anand, Christoph Borchers, Sébastien Brier, George M. Bou-Assaf, John R. Engen, S. Walter Englander, Johan Faber, Rachel Garlish, Patrick R. Griffin, Michael L. Gross, Miklos Guttman, Yoshitomo Hamuro, Albert J. R. Heck, Damian Houde, Roxana E. Iacob, Thomas J. D. Jørgensen, Igor A. Kaltashov, Judith P. Klinman, Lars Konermann, Petr Man, Leland Mayne, Bruce D. Pascal, Dana Reichmann, Mark Skehel, Joost Snijder, Timothy S. Strutzenberg, Eric S. Underbakke, Cornelia Wagner, Thomas E. Wales, Benjamin T. Walters, David D. Weis, Derek J. Wilson, Patrick L. Wintrode, Zhongqi Zhang, Jie Zheng, David C. Schriemer, Kasper D. Rand

**Affiliations:** 1 MRC Laboratory of Molecular Biology, Cambridge, UK; 2 Department of Biochemistry and Microbiology, University of Victoria, Victoria, BC Canada; 3 Department of Biochemistry, University of Colorado, Boulder, CO USA; 4 Department of Biological Science, National University of Singapore, Singapore, Singapore; 5 Genome BC Proteomics Centre, University of Victoria, Victoria, BC Canada; 6 Institut Pasteur, Chemistry and Structural Biology Department, Paris, France; 7 Biogen Idec, Cambridge, MA USA; 8 Department of Chemistry and Chemical Biology, Northeastern University, Boston, MA USA; 9 Department of Biochemistry and Biophysics, University of Pennsylvania, Philadelphia, PA USA; 10 Novo Nordisk, Måløv, Denmark; 11 UCB Celltech, Slough, UK; 12 Department of Integrative Structural and Computational Biology, Scripps Florida, The Scripps Research Institute, Jupiter, FL USA; 13 Department of Chemistry, Washington University in St. Louis, St. Louis, MO USA; 14 Department of Medicinal Chemistry, School of Pharmacy, University of Washington, Seattle, WA USA; 15 Johnson & Johnson Pharmaeutical Research and Development, Jersey City, NJ USA; 16 Bijvoet Center for Biomolecular Research, Utrecht University, Utrecht, the Netherlands; 17 Codiak Biosciences, Cambridge, MA USA; 18 Department of Biochemistry and Molecular Biology, University of Southern Denmark, Campusvej, Odense, Denmark; 19 Department of Chemistry, University of Massachusetts-Amherst, Amherst, MA USA; 20 Department of Chemistry, University of California, Berkeley, Berkeley, CA USA; 21 Department of Chemistry, The University of Western Ontario, London, ON Canada; 22 Institute of Microbiology, Czech Academy of Sciences, Prague, Czech Republic; 23 Department of Molecular Medicine, The Scripps Research Institute, Jupiter, FL USA; 24 Department of Biological Chemistry, The Hebrew University of Jerusalem, Jerusalem, Israel; 25 MRC Laboratory of Molecular Biology, Cambridge, UK; 26 Roy J. Carver Department of Biochemistry, Biophysics, and Molecular Biology, Iowa State University, Ames, IA USA; 27 Roche Innovation Center Penzberg, Penzberg, Germany; 28 Department of Early Stage Pharmaceutical Development, Genentech, Inc., South San Francisco, CA USA; 29 Department of Pharmaceutical Chemistry, University of Kansas, Lawrence, KS USA; 30 Department of Chemistry, York University, Toronto, ON Canada; 31 Department of Pharmaceutical Sciences, University of Maryland School of Pharmacy, Baltimore, MD USA; 32 Process Development, Amgen, Thousand Oaks, CA USA; 33 Department of Biochemistry & Molecular Biology, University of Calgary, Calgary, AB Canada; 34 Department of Pharmacy, University of Copenhagen, Copenhagen, Denmark

**Keywords:** Proteins, Biophysical methods, Biophysical chemistry, Mass spectrometry

## Abstract

Hydrogen deuterium exchange mass spectrometry (HDX-MS) is a powerful biophysical technique being increasingly applied to a wide variety of problems. As the HDX-MS community continues to grow, adoption of best practices in data collection, analysis, presentation and interpretation will greatly enhance the accessibility of this technique to nonspecialists. Here we provide recommendations arising from community discussions emerging out of the first International Conference on Hydrogen-Exchange Mass Spectrometry (IC-HDX; 2017). It is meant to represent both a consensus viewpoint and an opportunity to stimulate further additions and refinements as the field advances.

## Abstract (web summary)

Members of the hydrogen deuterium exchange mass spectrometry (HDX-MS) community provide their ‘best practices’ recommendations for HDX-MS data collection, analysis and reporting.

## Main

Hydrogen deuterium exchange mass spectrometry (HDX-MS) is a powerful technique that can provide insights into protein behavior by serving as a link between structure, conformational dynamics and function^1^. HDX-MS measures changes in mass associated with the isotopic exchange between amide hydrogens of the protein backbone and its surrounding solvent. The rate of this exchange is dependent on the folded state of the protein and its dynamics (particularly the stability of hydrogen bonding networks) and the intrinsic chemical properties of the underlying amino acid sequence^2–4^. HDX-MS is a very versatile technique^5^ and can be used to examine conformations of individual proteins or large protein complexes^6^, locate protein sites directly or indirectly involved in binding^7^, probe for allosteric effects^8^, monitor the folding dynamics of protein domains^9^, examine intrinsic disorder^10^ and provide insights into protein–membrane interactions^11^. Of particular relevance in industry, HDX-MS also excels at epitope mapping and the characterization of biotherapeutics^12,13^.

**Fig. 1:**
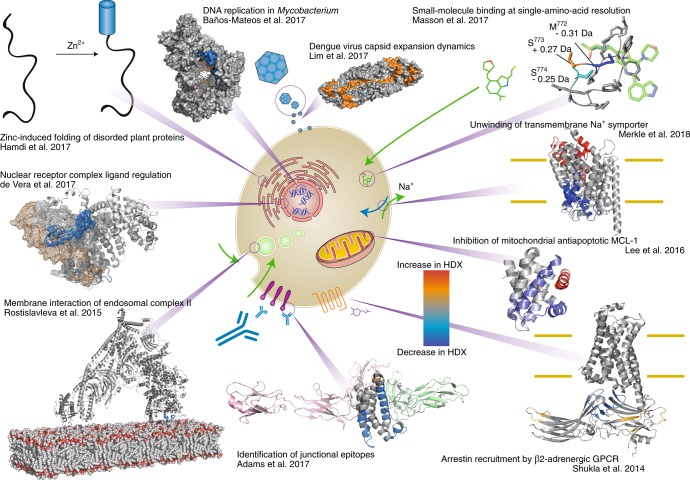
The wide range of applications for HDX-MS in many protein-folding studies. Clockwise from top left: Hamdi et al.^24^ localized dehydration and zinc-activated disorder-to-order transitions in abiotic plant stress response proteins using HDX-MS. Baños-Mateos et al.^25^ demonstrated how HDX-MS can be used in combination with X-ray crystallography and cryo-electron microscopy (cryo-EM) when determining the mechanism of exonuclease activity of DnaE1. Lim et al.^26^ determined how an increase in temperature alters the Dengue virus capsid structure, resulting in an alteration in antibody-binding mode. Using ETD-HDX/MS/MS, Masson et al.^27^ discerned the basis of isotype specificity of pharmaceutical compounds by determining single-residue exchange rates. Merkle et al.^28^ localized the substrate-dependent partial unwinding of transmembrane helices, which facilities substrate translocation using HDX-MS. Lee et al.^29^ revealed a new allosteric mechanism for interrupting the antiapoptotic binding of MCL-1 to BH3 domains, providing a new avenue for cancer therapy. Shukla et al.^30^ demonstrated how HDX-MS can provide mechanistic and dynamic detail to cryo-EM structures, and how HDX-MS can aid modeling of X-ray structures within the cryo-EM density. Adams et al.^31^ illustrated the utility of HDX-MS, used in conjunction with X-ray crystallography and biophysical methods, to reveal how the monoclonal antibody VHH6 contemporaneously interacts with IL-6 and gp80 through a junctional epitope. Rostislavleva et al.^32^ pushed the limits of HDX-MS with the large lipid kinase VPS34 complex II by both determining the membrane-interacting regions of the lipid kinase and screening nanobodies to facilitate crystallization and subsequent structure determination. By altering the pH of the labelling solution, de Vera et al.^33^ observed interactions of disordered protein domain on a millisecond timescale by HDX-MS.

Measurement of protein HDX dates back to the 1950s^14,15^, with much of the work in the 1970–1980s using NMR spectroscopy as the method of detection^16^. Mass spectrometry has been the method of choice for detecting HDX since the 1990s^17,18^, because it can accommodate large proteins (>100 kDa), accept low concentrations (less than micromolar) and tolerate complex sample matrices^19^. Additional advances in liquid chromatography (LC)-MS technology and automation have greatly increased the user base for HDX-MS. Data analysis software has progressed markedly^20–22^, increasing processing speeds by several orders of magnitude over the past ten years. The analysis of large multi-subunit proteins no longer takes weeks or months. A reduced barrier to entry has accompanied these developments, resulting in an explosion in the amount of HDX-MS data being generated. It is not surprising that the number and diversity of studies that use some variant of the HDX-MS method is increasing year over year^23–33^ (Fig. 1).

Producing a deuterium-labeled sample is simple and requires no specialized equipment or chemicals, other than D_2_O. However, the labeling reaction is highly sensitive to experimental conditions, such as pH and temperature fluctuations, among other factors^34^. It requires a number of controls and a great amount of care to ensure that the reaction is conducted in a manner that does not bias the results or lead to imprecise measurements. If properly performed, the HDX-MS method is very reproducible^7,35,36^, but repeated HDX reactions must be conducted to assess the degree of variability caused by external factors. When due care and attention is taken to fully account for all aspects of sample production^37^, HDX-MS provides reliable insights into protein structure–function properties.

HDX-MS data complement other structural biology techniques, such as nuclear magnetic resonance (NMR) spectroscopy^38^, electron microscopy^30,39^, native mass spectrometry^40,41^, molecular dynamics simulation^42^ and X-ray crystallography^43,44^, and are often presented alongside these techniques to nonspecialist reviewers. Likewise, scientists who primarily focus on these complementary techniques may, given the attractiveness and accessibility of HDX-MS, choose to embark on HDX-MS experiments themselves. Unfortunately, the simplicity of the experiment does not always translate into simplicity of interpretation, as the exchange mechanism is complex and important experimental information is all too often missing. Incomplete information compromises the ability to interpret published findings of others. To remedy this situation with several community-driven objectives, we aim to (1) develop consensus-based minimum recommendations for conducting HDX-MS experiments, along with the minimum information required for publication of HDX-MS data; (2) provide a resource for reviewers of manuscripts containing HDX-MS data, allowing them to assess the quality of presented data, and to assist editorial staff in establishing acceptable publication criteria; and (3) position the HDX-MS community for the creation of a standardized and curatable data format that allows for archiving datasets for meta-analysis and protein modeling.

## Development of the recommendations

The recommendations outlined below were initially discussed in May of 2017 during the first International Conference of Hydrogen Deuterium Exchange (IC-HDX) in Gothenberg, Sweden. The many HDX-MS experts present at IC-HDX debated the minimum criteria for HDX-MS experiments and manuscripts, and reached several consensus points. A draft document incorporating the primary elements of this debate was disseminated among the HDX-MS community, through discussion lists and through direct enquiries to experienced HDX-MS users who could not attend the meeting.

## Scope of the recommendations

These recommendations are precisely that: recommendations. They represent community-endorsed practices that ensure minimum requirements necessary to accurately conduct an HDX-MS experiment and report its results. Their aim is to be as practicable as possible to promote widespread adoption. Not all HDX-MS experiments need to conform to these recommendations, but in our opinion, any substantial deviation from these guidelines should be explained. Experiments that are purely technical, focusing on the minutiae of exchange behavior, or exchange under extreme conditions may deviate substantially from the recommendations, but these experiments are likely to be conducted by those already expert in the technique, with an explanation of their reasoning for selecting these conditions.

We also recommend that newcomers to HDX-MS familiarize themselves with the theoretical basis of hydrogen exchange and how protein structure influences this phenomenon. An appreciation of this theoretical background is crucial for the interpretation of HDX-MS data, and much literature exists on the subject^1,2,19^.

**Fig. 2:**
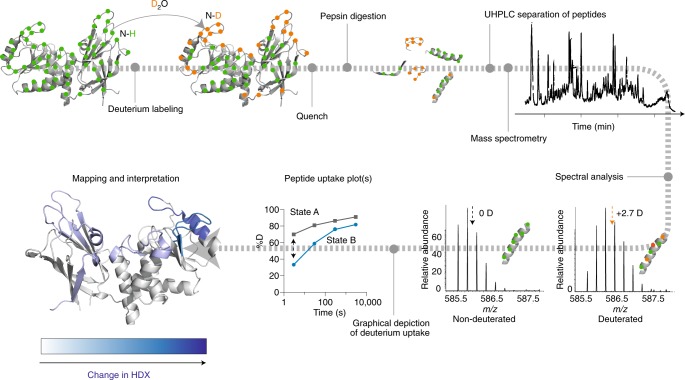
The common ‘bottom-up’ or ‘local’ HDX-MS experiment. Proteins are incubated in deuterated buffer for a number of time points, allowing for the incorporation of deuterium into the protein backbone. The exchange reaction is quenched by a shift to acidic pH and a temperature drop (with the optional inclusion of denaturants and reducing agents to enhance protein unfolding). Proteins are then digested by an acid-functional protease, such as pepsin. The proteolytic peptides are desalted and separated using a chilled reversed-phase UHPLC system and eluted into a mass spectrometer, where they are ionized by electrospray and subjected to mass analysis to determine the increase in mass resulting from deuterium uptake. During spectral analysis, the isotopic envelopes of peptides are visualized, and levels of deuteration are determined, typically through comparison of the average mass from the intensity-weighted centroid *m/z* value (arrows) of the peptide. The example mass spectra show that the peptide has a deuterium level of 2.7 D. The deuterium uptake, resolved to individual peptide segments, is plotted across multiple time points. Peptide uptake plots reveal the local HDX profile of individual protein regions. Peptide uptake plots obtained in an identical manner for multiple states of the protein, such as folded and unfolded, or bound and unbound to a ligand, can be overlaid to enable quick comparison and detection of local differences in HDX (and conformation) between protein states. Such differences in HDX can then be mapped on a three-dimensional representation of the protein to facilitate structural interpretation. Structure adapted from Lee et al.^58^.

Currently, the most widely used HDX-MS experimental format involves an in-solution labeling step followed by injection onto an immobilized protease column, desalting and separation of proteolytic peptides using a cooled reversed-phase LC system and finally mass analysis conducted by a mass spectrometer (Fig. 2). This classical continuous-labeling, bottom-up or local HDX-MS^17–19,45^ experiment is the focus of the current recommendations, but it is by no means the only HDX-MS experiment. In the future we may address other variations such as global HDX-MS analysis^45^, bottom-up or top-down HDX-MS/MS^46–49^ and gas-phase HDX-MS^50^. The two primary issues we wish to address for classical bottom-up HDX MS experiments are reproducibility and transparency.

### Reproducibility

To produce quality studies, it is necessary to ensure that, given the same materials, instrumentation and protocol, the same observations would be made for any given experiment. This requirement impacts sample production, data acquisition and analysis.

### Transparency

Data need to be more readily accessible to the community. These recommendations aim to create conduits to raw data, enabling further meta-analysis, providing increased confidence in reported observations and supporting ongoing software development efforts.

If the recommendations are followed, we believe the end result will be an overall increase in the quality of published data and more meaningful scientific insights gleaned from the findings. These recommendations may require revision, enhancement and additions in the future as HDX-MS technology advances and evolves. At each future IC-HDX meeting, the current recommendations should be assessed, and altered or revised as necessary to keep pace with this fast-moving field.

1. **Sample preparation and analysis recommendations**
  11. We recommend that a sample quality assessment precede the HDX experiment. The assessment could include denaturing electrophoresis (SDS-PAGE) analysis and intact protein mass spectra to confirm sample purity and confirm the expected sequence and post-tranlational modicications. A size-exclusion chromatography or native MS analysis is useful to establish the monomeric or oligomeric state of the sample being investigated, or a functional biochemical assay to check that the protein is active and correctly folded.
  12. We recommend a size-exclusion chromatography analysis to establish the monomeric/oligomeric state of the sample being investigated.
  13. Sample preparation is key to a reliable HDX-MS experiment. Given the sensitivity of HDX-MS, any perturbations in pH (or pD), temperature and ionic strength will have considerable effects on the outcome of any experiment, and so it is crucial to control these parameters. At a minimum, the buffer used in the labeling reaction must have sufficient buffering capacity to ensure a constant pH, and the temperature of the labeling reaction must be well controlled. Therefore, the composition of the buffer used in the labeling reaction and the temperature and pH_read_ (pH meter reading with no isotope corrections applied) at which the reaction was conducted must be reported. Both labeling buffer and protein solution must be pre-equilibrated at the temperature of the ensuing HDX experiment and stably maintained during labeling.
  14. The concentration of D_2_O (%, v/v) present during the labeling reaction must be precisely maintained and clearly reported. HDX experiments may be conducted with any concentration of D_2_O, but experiments are typically conducted at higher concentrations of D_2_O (80−90%), as this leads to greater deuterium incorporation (resulting in a larger mass shift). Achieving the highest signal-to-noise ratio (that is, sensitivity to distinguish differences in HDX between protein states) and minimizing spectral complexity can require optimizing the concentration of D_2_O used^51^, which should be precisely maintained throughout the experiments and reported.
  15. Quench buffer composition greatly affects the efficiency of digestion, and thus the protocol used for quenching should be reported (composition and pH of the quench buffer). The final composition of the quenched sample, that is, the concentration of labeled protein and quench solutions, as well as the pH of the quenched sample, should also be reported.
  16. Repeated measurements of deuterium incorporation are necessary to ensure repeatability and deliver an estimate of the precision in the measurements. Independently generated exchange reactions serve as technical replicates. The same labeling reaction aliquoted and measured separately is not a suitable technical replicate, as this is not an independent observation. At a minimum, there should be at least three labeling reaction experiments performed for at least one time point to allow a reasonable estimate of the error of measured deuterium levels. This estimate of error should be used to support the assignment of significance to differences in HDX between states. Labeling reactions (more than six) performed with extraction of many samples across a wide time range (more than four orders of magnitude) can provide additional confidence in the assignment of statistically significant differences in HDX. Where practical issues arise (for example, restricted sample supply), replicate measurement may not be necessary for all the time points in the reported HDX curves.
  17. Biological replicates of the experiment should be conducted where possible. This would require additional preparations of the protein. The repeats ensure that the variability in exchange measurements that can be ascribed to post-translational modifications/differences in protein expression/purification or variable stoichiometry in reconstituted protein complexes is quantified. Biological replicates are especially important for proteins that require extensive sample preparation before HDX-MS (for example, nucleotide loading in monomeric GTPases^52^).
  18. The LC-MS system used to collect the data should be made explicit in the methods section of the manuscript, along with pertinent instrument settings (for example, the LC gradient and flow rates, reversed-phase columns used, MS ion source parameters and so on).
  19. As a variety of proteases are available for sample workup, the protease used in the experiment should be stated. Additionally, the duration of the digestion, the digestion mode (off-line or on-line) and the temperature that the digestion was conducted under should be reported. In the case of on-line digestion, column dimensions, source and flow rate should be specified.
  20. Evidence of LC-MS system suitability for reproducible deuterium recovery is required. The level of back-exchange (loss of deuterium) of the particular HDX-MS system and workflow being used must be characterized in detail by analysis of a mixture of model peptides (for example, bradykinin or angiotensin II) or ideally a digest of a model protein (for example, hemoglobin, phosphorylase B or cytochrome c) that has been equilibrated in deuterated buffer for an extended time period (for example, 12 h) to allow complete labeling of all backbone amide NHs in the model peptide/protein. If such a characterization of the workflow and LC-MS system in use has been performed in an earlier study, that work can be referenced. The characterization serves to validate the workflow and LC-MS setup and ensures that a suitable level of deuterium is retained during any given experiment. The back-exchange of peptides of the model system used can be calculated using the following equation^18,45,53^:
    $${\mathrm{Back}}\;{\mathrm{exchange}} = \left( {1 - \frac{{m_{100\% } - m_{0\% }}}{{{{N}} \times {{D}}_{{\mathrm{frac}}}}}} \right) \times 100$$
    where *m*_0%_ is the non-deuterated peptide centroid mass, *m*_100%_ is the maximally labeled peptide centroid mass, *N* is the theoretical number of backbone amides in the peptide and *D*_frac_ is the fraction of *D*/*H* in the labeling buffer used (for example, 0.80, 0.90, 0.95). Back-exchange levels are ideally reported on a per-peptide basis but may be reported as the average percentile loss of deuterium of all peptides analyzed with an indication of the range of values observed, for example, 40% (ranging from 10% to 55%). In a well-conducted conventional state-of-the-art bottom-up HDX-MS workflow, only very few peptides should exhibit back-exchange values above 50%.
  21. We recommend producing a ‘maximally labeled’ control sample of the protein studied (also known as a 100% exchange control), particularly in situations where the absolute amount of exchange is desired. This control allows for an estimate of the level of back-exchange for each analyzed peptide during sample work-up and analysis. A ‘quench exchange’ control sample may also be used to estimate the amount of on-exchange that occurs during the quench process, but unless long quench/digestion times (> min) or non-ideal quench conditions (pH > 2.5) are required, this is usually well-approximated by the non-deuterated centroid mass in a typical bottom-up LC-MS experiment. From this information, a back-exchange corrected fractional deuterium level (*D*_corr_) can be estimated using the following equation:
    $$D_{\mathrm{corr}} = \frac{{\left( {m - m_{0\% }} \right)}}{{\left( {m_{100\% } - m_{0\% }} \right)}}$$
    where *m* is the observed peptide centroid mass at a given time-point, *m*_0%_ is the non-deuterated peptide centroid mass and *m*_100%_ is the maximally labeled peptide centroid mass. When *D*_corr_ is expressed as a percentage value, it is sometimes referred to simply as the ‘back-exchange’ corrected percentage *D* value for a given analyzed peptide. A more explicit treatment can be found in ref. ^53^.
    The absolute amount of exchange (*D*_absolute_) in the peptide can then be calculated based on *D*_corr_ as *D*_absolute_ = *D*_corr_ × *N*, where *N* is the theoretical number of backbone amides in the peptide. The absolute amount of exchange can be required for interpreting HDX-MS data in a structural context, for example, for identifying intrinsically disordered regions. These controls are not strictly necessary for comparative measurements between different states of the same protein (for example, with and without ligand), as the back-exchange can reasonably be expected to be the same with each measurement. Furthermore, we recommend performing the labeling reaction that produces the maximally labeled control sample for 12−24 h at room temperature and low pH (2.5 < pH < 4) in the presence of a strong denaturant (for example, 6 M GndDCl or 6 M urea). Such a treatment usually offers adequate HDX equilibration between protein and labeling solution ensuring complete labeling of all backbone amide NHs. However, in rare cases, a maximally labeled control prepared in this manner can fail to exchange a minor subpopulation of very slow exchanging amide NHs, and furthermore sample aggregation can also be a concern for some proteins^21^. Preparation of maximally labeled control samples at higher pH values (pH > 5) and excessive labeling times (>24 h) or elevated temperatures (>25 °C) should be approached with caution, as histidine residues may, at these conditions, begin to incorporate a substantial amount of deuterium at the imidazole sidechain (c-2 position), leading to higher than expected levels of deuterium^54^. In any case, a carefully prepared, maximally labeled control sample of the target protein under study will serve as the best possible estimate of the maximal level of deuterium one can expect to detect in each peptide analyzed during the given HDX-MS experiment.
  22. A wide range of D_2_O labeling times should be used to interrogate the full range of possible amide exchange rates^39^. We recommend that labeling times span at least four orders of magnitude (for example, 0.1 min, 1 min, 10 min, 100 min and 1,000 min), with the shorter times in the range of 5−15 s, and the longer times lasting at least several hours. Importantly, sampling labeling times beyond four orders of magnitude can provide additional useful information on HDX kinetics and error (see also recommendation 1.5). The exact time range will depend on the protein system in question. For example, experiments on intrinsically disordered proteins, which are likely to exhibit rapid HDX rates, can focus on even shorter labeling times (that is, 0.1 s, 1 s, 10 s or 100 s). For very stable proteins that may have extensive regions that undergo very slow HDX, experiments should be performed with very long labeling times (>1,000 min or 16 h). The selection of time points should be justified in the manuscript; it is important that the time points cover a range sufficient to allow for a substantial change in the deuterium level of the majority of the amides in a protein, while still allowing for the detection of fast exchange events. In short, the range should be targeted to the nature of the investigation, such as shorter labeling times with narrower time ranges for investigating transient molecular interactions or disordered proteins. Selecting the labeling times is particularly important when designing appropriate null hypothesis experiments for differential HDX studies (that is, failing to disprove that there is no difference in HDX between two conditions).
  23. Especially for longer labeling times (for example, over 100 min), we recommend conducting a quality control ‘deuterium-pulse’ experiment if the physical stability of the protein of study is unknown under labeling conditions. In such an experiment, the protein is incubated for a time equal to that of the longest labeling time but in the absence of deuterium and is then deuterium-labeled for a short time period (for example, 10−30 s). This pulse-labeled sample should then be compared to the sample from the equivalent short-labeling experiment but without the prior incubation. The purpose of this comparison is to check whether the protein is undergoing structural changes (for example, precipitation, oligomerization or irreversible unfolding) over the course of the labeling time, which may result in misinterpretation of data.
  24. To ensure optimal sensitivity for detecting changes in HDX during ligand-binding experiments, we recommend optimizing ligand concentrations and ensuring adequate time for complex formation to ensure maximum protein occupancy. Ligand and protein concentrations used during labeling should be stated, as well as the dissociation constant (if known).
  25. In comparative HDX-MS analyses, we recommend following good practices in experiment design to control variation. Analysis should be randomized. Our recommendation is to avoid collecting data for all states sequentially or collecting technical replicates sequentially for one state. By doing so, one can mitigate the effects of any drifts in instrument parameters, day-to-day variability (in case data are collected over multiple days), temperature fluctuations or any other parameter that might affect the HDX-MS experiment.
2. **Data analysis and data presentation guidelines**
  21. When conducting peptide identification using MS/MS, the spectral search database should include the sequence of all major proteins present in the sample and introduced during workup, to prevent false peptide identifications. This is especially advisable for complex sample types. The list should minimally include the proteases used and any major protein contaminants. The composition of the database used for searching should be reported.
  22. Peptide identification criteria should be included in the text, based on the search tool used (that is, scoring cut-off and its statistical basis). The name of the search software (and version) should be provided, with parameters appropriate for the mass spectrometer used.
  23. For quality control purposes, the output of automated HDX-MS computational routines should be supported by an inspection of the raw data, including spectral assignments and isotopologue detection. A summary of the HDX data should be reported in a table (for example, Table 1) with the following information for each protein included within the study: (1) HDX reaction details, for example, pH and temperature; (2) HDX time course, for example, what time points where analyzed; (3) number of peptides analyzed (that is, the total number of peptides for which the deuterium content has been analyzed in each dataset); (4) sequence coverage, expressed as a percentage of amides covered by the peptides for which deuterium content has been measured (rather than all peptides identified in the non-deuterated experiment); (5) average peptide length and redundancy; (6) a quantitative measure of the repeatability of deuterium measurement, for instance, the average (mean, median, root-mean-square) standard deviation from replicate (technical or biological) measurements of the deuterium content of all or representative peptides from one or more time points; and (7) threshold for significant differences in HDX (a threshold value interpreted as representing a significant difference in HDX between examined protein states based on the quantitative measure of repeatability. We recommend that such a table be provided in the supplementary material for all HDX manuscripts, similar to the convention in the X-ray crystallography field of reporting collection/refinement statistics^55^. We include an example of the HDX summary table as a downloadable template spreadsheet (Supplementary Table 1) to encourage the community to include such data in their reporting, and to do so using a standardized and readily accessible format. We also recommend that a peptide coverage map—a figure showing the identified peptides used to extract HDX information mapped onto the sequence of the protein studied—be included in the manuscript or in the supplementary material.

**Table 1:** HDX data summary

Dataset	Protein state (one column for each condition: that is, apo, ligand-bound, mutant and others)
HDX reaction details	Labeling conditions, for example, percent D_2_O, pH_(read)_, temperature and so on
HDX time course	Listing of what time points were analyzed
HDX controls	Description of HDX control samples analyzed
Back-exchange	Back-exchange (average) for all peptides measured (model system or studied protein) and the interquartile range of these values
Number of peptides	Description of the number of peptides used for which HDX data were obtained
Sequence coverage	Expressed as the percentage of amides covered by the peptides for which HDX data were obtained
Average peptide length/redundancy	Average peptide length and number of readings for any amide (calculated as the total number of peptides for which HDX data were obtained over the total number of amides)
Replicates (biological or technical)	Number and specification of replicate HDX-MS measurements performed for each condition and deuterium incorporation time point
Repeatability	A quantitative measure of the repeatability of deuterium measurement (for example, the average standard deviation from technical replicate measurements of the deuterium content of all peptides from one or more time points for a single condition)
Significant differences in HDX	A value used as a threshold to represent a significant difference in HDX between examined protein states as based on a quantitative measure of repeatability
  24. When reporting explicitly on the change of HDX in a peptide owing to, for example, the presence of a binding partner, a peptide uptake plot should be provided, plotting each labeling time with the per-peptide standard deviation. Appropriate statistical analyses should be applied to all reports of differences between states. Furthermore, HDX information from multiple charge states and overlapping peptides should be used to add certainty to any conclusions.
  25. A common data presentation format for HDX-MS data involves color-mapping the time-resolved, peptide-resolution data onto three-dimensional, static, atomic-resolution structures. Inappropriate mapping can result in a loss of information. When mapping HDX data onto structures, scientists should explicitly state their mapping methodology and at which time points data are depicted, and this approach should be based on a quantitative and statistical argument that is applied to the entire dataset. Such an encompassing method avoids selective presentation and permits a balanced assessment of biologically relevant findings. In cases where a difference in HDX is mapped, regions should be carefully indicated for which there is no sequence coverage. Furthermore, regions that exhibit ‘no significant difference’ should not be interpreted as ‘no change’. Rather, it means no detectable difference within the kinetic regime had been detected. The mapping should be accompanied with an explicit statement of this nature. Finally, both authors and readers must recognize that imposing HDX-MS results (measured in a dynamic solution-phase environment) on static structures can easily bias interpretation of the results. Two very different biophysical measurements are being combined, so it is difficult to convey the magnitude of conformational change on a single static structure. Interpretation is most accurate when based on HDX uptake plots.
3. **Supplemental data presentation recommendations**
  31. A supplementary ‘HDX data’ table corresponding to all the peptides included in the study, including peptides that show no significant difference between states, should be presented. Two examples of such a table are shown in Supplementary Tables 2 and 3; either can be used depending on the output format of the HDX-MS data analysis software in use. We include these downloadable template spreadsheets to encourage the community to include such data in their reporting and to do so using this standardized and readily accessible format. The HDX data table greatly simplifies access to the acquired data for other scientists and will enable any downstream data processing, including use of the data for computational modeling. The minimum requirements are peptide sequence (start and end numbering and sequence), peptide monoisotopic mass (uncharged), chromatographic retention time, mean deuterium uptake (shift in average/centroid mass, without any correction for back-exchange) and the standard deviation, for each labeling time, with a clear indication of the number (*n*) and nature of replicates (technical/biological) used to determine this value. These data should be provided for all states measured (for example, apo and with a binding partner). If a maximally labeled control and quench exchange control samples are analyzed, similar data should be reported for these as well.
  32. Provided that an HDX summary table (Table 1) and an HDX data table (Supplementary Tables 2 and 3) are provided, only deuterium uptake plots for peptides that are explicitly referenced within the main body of the manuscript are necessary to include (in the manuscript or supplementary information). These plots should show the appropriate deuteration values across all measured labeling times, with standard deviations between technical repeats indicated.
  33. For peptides undergoing substantial EX1 or mixed exchange profiles^56^, we recommend the inclusion of raw deuterium incorporation mass spectra, or the use of deuterium incorporation plots that show the different bimodal populations (bubble plots can be a useful data representation for this type of data; see ref. ^57^).

## Conclusion

The objective of our recommendations is to increase the transparency and utility of HDX-MS analyses for the reader, the wider community, the reviewer and the experimentalist. We recognize that these recommendations are not exhaustive and should be viewed as a consensus-based first iteration of a continuously evolving system of community self-review. One of the most pressing future steps is to agree to a standardized data structure that can be archived, retrieved and annotated on an open data server. There is no agreed-upon mechanism for making published data readily available to interested parties, and we are not entirely convinced that co-opting proteomics repositories is the best strategy. As a first step to address this issue, we recommend including the ‘HDX summary’ table and also the ‘HDX data’ table as described here, in future publications of HDX-MS studies. We recommend including the following sentence in methods section of all papers: “To allow access to the HDX data of this study, the HDX data summary table (Table X) and the HDX data table (Table Y) are included in the supporting information as per consensus guidelines (with a reference to this manuscript).” We also recommend that raw HDX-MS data be made available for reanalysis to interested parties through personal file sharing whenever possible, until a standardized structure for raw data and an open data server for annotated HDX-MS data have been agreed upon or established by the community.

The development of a standardized approach for reporting of HDX data has major advantages in increasing the quality of published HDX-MS data. First, the current disparity in published manuscripts as to what data are presented, and how these data are presented, can make reviewing and assessing HDX-MS experiments challenging. Second, certain key statistics and metrics that may affect the validity of the publication’s conclusions are sometimes reported in supplementary data or not mentioned at all. Third, there are enormous unexplored opportunities associated with meta-analyses of archived HDX-MS data. Access to large sets of raw data could provide insights into the mechanism underlying the HDX process—a phenomenon still not completely understood—and furthermore improve the utility of HDX-MS data for computational modeling of protein structures. Finally, a shared database would avoid unnecessary repetition of experiments. We believe that our recommendation to include both ‘HDX summary’ and ‘HDX data’ tables as described above would greatly facilitate upload and storage of HDX-MS data in a central community-accessible database.

## Supplementary information

Supplementary Table 1 HDX summary table. A template spreadsheet for reporting critical HDX-MS information.

Supplementary Table 2 HDX data table. Example 1 of data output for kinetics experiments, to be used as a template.

Supplementary Table 3 HDX data table. Example 2 of data output for kinetics experiments, to be used as a template.
